# Renal Embolism Associated with Foramen Ovale Coexisting Acute Pulmonary Embolism

**DOI:** 10.1155/2023/6670080

**Published:** 2023-12-06

**Authors:** Yanling He, Yi Xiao, Yanping Chen, Zhidong Li

**Affiliations:** Yan'an Hospital Affiliated to Kunming Medical University, China

## Abstract

We report a singular case of renal embolism in a hitherto healthy 46-year-old female. The patient initially presented with symptoms of exertional distress and chest discomfort. Following an extensive diagnostic workup, she was subsequently diagnosed with acute pulmonary embolism. On the day succeeding her admission, the patient manifested sustained abdominal discomfort. Abdominal computed tomography angiography (CTA) subsequently revealed the presence of renal artery embolisms and infarctions. Concurrently, an echocardiographic evaluation disclosed a patent foramen ovale (PFO) and pulmonary hypertension. In this specific case, we hypothesize that the embolic event traversed through the PFO, ultimately localizing in the renal artery and culminating in renal embolism.

## 1. Introduction

Pulmonary embolism (PE) ranks as the third leading cause of cardiovascular-related deaths worldwide, superseded only by stroke and myocardial infarction [1]. The embolism often originates from deep vein thrombosis (DVT) [2]. Additionally, the embolism can traverse from any location for various reasons; this scenario is termed paradoxical embolism (PDE). Paradoxical embolism (PDE) occurs when a thrombus crosses an intracardiac defect into the systemic circulation [3, 4]. One etiological factor is the patent foramen ovale (PFO). The foramen ovale is a flap-like communication between the right and left atria at the level of the fossa ovalis. This communication usually closes after birth due to increased pressure in the left-sided cardiac cavities associated with normal breathing. If the foramen persists beyond the age of one [5], it is termed a patent foramen ovale (PFO). While most individuals with PFO remain asymptomatic, some may experience hypoxemia, platypnea–orthodeoxia syndrome, and neck pain [6, 7, 8]. A peculiar anomaly, the patent foramen ovale (PFO), facilitates a detour of pulmonary circulation by shunting the right-sided venous circulation into the left-sided arterial system [9]. This diversion instigates a paradoxical embolism, defined as a systemic arterial embolism prompted by the ingress of venous thrombi into the arterial circulatory system via a right-to-left shunt [10]. The transit of a thrombus through a patent foramen ovale (PFO) in a PE patient is an infrequent phenomenon, often coupled with high mortality rates [11]. Several retrospective and prospective observational studies have indicated a high prevalence of stroke among patients with PE and a disproportionately high prevalence of PFO in patients with PE who experienced a stroke [12, 13, 14, 15]. The underlying mechanism is attributed to paradoxical embolism. Other causes include “in situ” thrombosis and dysrhythmia [16]. A PE-associated renal embolism amplifies the suspicion of a paradoxical embolic pathway, which can be corroborated by an echocardiographic display of a thrombus straddling a patent foramen ovale. However, even in instances lacking this diagnostic hallmark, a paradoxical embolism diagnosis can still be established [17]. Concurrent occurrences of pulmonary thromboembolisms (PTE) and deep vein thromboses (DVT) are not uncommon and have been extensively documented [18, 19]. Rare incidents of arterial systemic embolic involvement through PEO have been reported. We hereby present an unusual case of renal artery embolism instigated by a PDE via PFO, which was further complicated by extensive PTE, DVT, and renal infarctions.

## 2. Case

**Figure 1:**
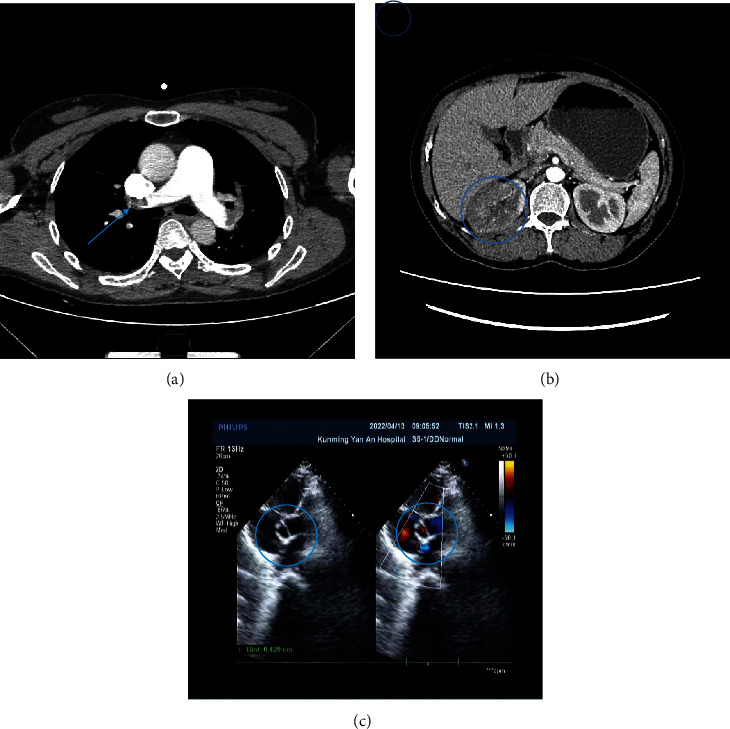
(a) Thoracic computed tomography depicts pulmonary embolism. (b) Renal ischemia and infarction as illustrated by abdominal computed tomography. (c) Patent foramen ovale, as evidenced by echocardiography.

A 46-year-old female patient (domestic cleaner), manifesting dyspnea upon exertion and chest discomfort for one day. Prior to her discomfort, she was resting at home. She was admitted to our hospital in April. No evidence of syncope or any other symptoms was presented. The patient was diagnosed with adenomyosis but was not under any treatment regime. Her prior medical, medication, and family histories were insignificant, and she was a nonsmoker. Physical examination revealed a temperature of 36.2°C, a blood pressure reading of 95/78 mmHg, and a pulse rate of 82 bpm. Her pulmonary auscultation displayed moist rales, and her heart sounds were muted with no observable murmurs. Palpation of the abdomen and neurological assessments appeared normal. For younger patients with dyspnea upon exertion and chest discomfort, respiratory and cardiovascular diseases are often considered, but she had no relevant history. She could lie down, and her heart auscultation was almost normal, leading us to suspect an acute condition, such as severe pneumonia. While her blood test results were largely normal, a significantly elevated D-dimer level necessitated a compression ultrasonography and a computed tomographic pulmonary angiography. These tests confirmed a DVT and a massive PTE, depicted by thromboembolism in both the left and right pulmonary arteries and a left peroneal vein thrombosis (Figure 1(a)). Concurrently, to preemptively detect heart failure and pulmonary hypertension, an echocardiogram was conducted, which unveiled a PFO (Figure 1(c)) and pulmonary hypertension (reaching 80 mmHg). The day after admission, the patient experienced abdominal discomfort, which worsened to persistent abdominal stress pain in the following day. There was no tenderness, rebound tenderness, or muscular tension throughout the abdomen. A routine urine test revealed erythrocytes, although the patient was not menstruating. An ensuing abdominal CT angiogram disclosed renal artery embolisms and infarctions (Figure 1(b)). This discovery was missed in the initial CT angiogram conducted during the diagnosis of the pulmonary embolism. The ensuing renal infarctions and embolisms were attributed to the acute PE-induced continuous opening of the PFO, which enabled thrombi from the deep vein to enter the arterial system. The confirmed diagnosis introduced a therapeutic challenge, as treatment varies depending on whether the embolism occurs in the vein or artery. Aspirin is often the treatment of choice for arterial embolisms, whereas rivaroxaban is preferred for venous embolisms. Administering both carries a significant risk of critical organ hemorrhages, such as cerebral hemorrhages. Several case reports in the literature reflect similar predicaments [20, 21]. Ultimately, we opted for anticoagulation therapy using only rivaroxaban. A month later, a subsequent CT scan showed improvement.

## 3. Discussion

The foramen ovale is an important fetal structure that closes after birth in most individuals and remains open as a patent foramen ovale (PFO) in approximately 25% of the healthy population [22]. A thrombus through a PFO with impending paradoxical embolism is an extremely rare event [23, 24]. Most renal infarctions are related to infective endocarditis, atrial fibrillation, and renal artery dissection [25, 26, 27]. This patient did not have the relevant diseases or any past history. Her complaints with physical examination and testing together support any of these possible diseases. In this condition, there is considerable evidence suggesting that PFO is highly associated with renal artery embolism, and PFO detection is very important and necessary. There are some cases of cryptogenic stroke with PFO and PTE [28, 29, 30, 31], but little cases with renal artery embolism and renal infarction [32, 33]. In this case, there was a renal artery embolism and infarction, and there is no evidence of coagulopathy, which can cause the systematic embolism. Although we did not catch the embolism straddling through the PFO, the mechanism of renal embolism and infarction in our case considered that the opening of the PFO permitted a passage for thrombi to travel in the arterial system. This patient has abdomen stress pain that indicates with renal infarction at last, but the patient with silent thromboembolisms of PFO should require a systematic search to trace progress and infarction. According to Lacut et al.'s study [34], there is a higher risk of ischemic stroke in PE patients with PFO compared to those without PFO, with a 73% likelihood that PFO is the mechanism of stroke in these patients [35]. Therefore, patients should undergo a cerebral MRI to identify any potential embolism. Although this patient did not undergo cerebral MRI due to cost and lack of related symptoms, such imaging is advisable where possible to rule out microinfarction.

In conclusion, if acute organ infarction cannot be explained in thrombosis or traditional embolism, the PFO should be considered. Patients with silent thromboembolisms related to PFO should warrant a systematic investigation to track progression and infarction. For patients with both venous and arterial embolisms, administering rivaroxaban alone is sufficient and effective.

All the participants were accepted for this experiment, and informed consent was obtained from all individual participants included in the study.
